# Estrogen receptor alpha/beta ratio and estrogen receptor beta as predictors of endocrine therapy responsiveness–a randomized neoadjuvant trial comparison between anastrozole and tamoxifen for the treatment of postmenopausal breast cancer

**DOI:** 10.1186/1471-2407-13-425

**Published:** 2013-09-18

**Authors:** Marcelo Madeira, André Mattar, Ângela Flávia Logullo, Fernando Augusto Soares, Luiz Henrique Gebrim

**Affiliations:** 1 Senology Discipline, Department of Gynecology, Federal University of Sao Paulo-UNIFESP, R. Botucatu, 740, 04023-900 Sao Paulo, SP, Brazil; 2 Department of Obstetrics & Gynecology and Women’s Health of Albert Einstein Hospital, Av. Albert Einstein, 627, 05652-900 Sao Paulo, SP, Brazil; 3 Department of Breast Medical Oncology, Centro de Referência da Saúde da Mulher (CRSM)-Pérola Byington Hospital, Av. Brigadeiro Luis Antonio, 683, 01317-000 Sao Paulo, SP, Brazil; 4 Department of Pathology, Federal University of Sao Paulo-UNIFESP, R. Botucatu, 740, 04023-062 Sao Paulo, SP, Brazil; 5 Department of Pathology, AC Camargo Hospital, R. Professor Antonio Prudente, 211, 01509-010 Sao Paulo, SP, Brazil

**Keywords:** Estrogen receptor beta, Breast cancer, Estrogen receptor, Aromatase inhibitors/Anastrozole, Tamoxifen, Ki67, Neoadjuvant therapy, Tumor markers

## Abstract

**Background:**

The role of estrogen receptor beta (ER-β) in breast cancer (BC) remains unclear. Some studies have suggested that ER-β may oppose the actions of estrogen receptor alpha (ER-α), and clinical evidence has indicated that the loss of ER-β expression is associated with a poor prognosis and resistance to endocrine therapy. The objective of the present study was to determine the role of ER-β and the ER-α/ER-β ratio in predicting the response to endocrine therapy and whether different regimens have any effect on ER-β expression levels.

**Methods:**

Ninety postmenopausal patients with primary BC were recruited for a short-term double-blinded randomized prospective controlled study. To determine tumor cell proliferation, we measured the expression of Ki67 in tumor biopsy samples taken before and after 26 days of treatment with anastrozole 1 mg/day (N = 25), tamoxifen 20 mg/day (N = 24) or placebo (N = 29) of 78 participants. The pre- and post-samples were placed in tissue microarray blocks and submitted for immunohistochemical assay. Biomarker statuses (ER-β, ER-α and Ki67) were obtained by comparing each immunohistochemical evaluation of the pre- and post-surgery samples using the semi-quantitative Allred’s method. Statistical analyses were performed using an ANOVA and Spearman’s correlation coefficient tests, with significance at p ≤ 0.05.

**Results:**

The frequency of ER-β expression did not change after treatment (p = 0.33). There were no significant changes in Ki67 levels in ER-β-negative cases (p = 0.45), but in the ER-β-positive cases, the anastrozole (p = 0.01) and tamoxifen groups (p = 0.04) presented a significant reduction in post-treatment Ki67 scores. There was a weak but positive correlation between the ER-α and ER-β expression levels. Only patients with an ER-α/ER-β expression ratio between 1 and 1.5 demonstrated significant differences in Ki67 levels after treatment with anastrozole (p = 0.005) and tamoxifen (p = 0.026).

**Conclusions:**

Our results provide additional data that indicate that the measurement of ER-β in BC patients may help predict tamoxifen and anastrozole responsiveness in the neoadjuvant setting. These effects of hormonal treatment appear to be dependent on the ratio of ER-α/ER-β expression.

**Trial registration:**

Current Controlled Trials ISRCTN89801719

## Background

Breast cancer (BC) is the most frequently diagnosed non-skin cancer among women worldwide [1-3]. The survival rate at 5 years after diagnosis in the United States has improved from 63% in the early 1960s to 89% currently [3]. Adjuvant hormone therapy has helped achieve this substantial reduction in mortality because approximately 75% of human BCs express estrogen receptors (ERs) [4-6].

Estrogens play a central role in the development and growth of both normal and malignant mammary tissues. In addition, they mediate most of their action through the alpha ER (ER-α) [7]. Pathological lesions associated with increased risk of BC also present significantly more cells expressing ERs [8]. The ER-α status of breast tumors provides prognostic information and is the primary target for endocrine therapy. Effective strategies to treat ER-positive BC include endocrine agents that compete with estrogen for binding to its receptor, such as selective estrogen-receptor modulators (SERMs) and antiestrogens or reducing the levels of circulating estrogens by the administration of agents such as third-generation aromatase inhibitors (AIs), which have been shown to be more effective than tamoxifen in postmenopausal women in neoadjuvant and adjuvant settings [9].

The discovery in 1996 [10] of a second ER subtype, known as beta (ER-β), which presented different expression profiles in normal and malignant tissues, opened the possibility that breast tumors might be even more heterogeneous than originally thought. The role of ER-β in BC initiation and development has not yet been clearly established [11]. *In vitro* experiments have demonstrated that ER-β inhibits the proliferation, migration and invasion of BC cells [12-15] and the angiogenesis and growth of tumor xenografts [16]. The potential clinical use of ER-β in BC endocrine therapy has been investigated in retrospective studies, and ER-β positivity has been associated with significantly better survival in patients with ER-α-negative, progesterone receptor (PgR)-negative and triple-negative tumors treated with adjuvant tamoxifen therapy. These types of tumors are widely believed to be hormone unresponsive [17].

Despite initial positive responses to tamoxifen therapy, one-third of all patients will develop resistance, though their ER-α status may remain unchanged [18,19]. A lower expression of ER-β is found in tamoxifen-resistant tumors, and high levels of ER-β are occasionally associated with a better clinical outcome in ER-α-positive breast tumors [11]. Several studies have suggested that the expression of ER-β independently predicts a better disease-free survival in patients treated with tamoxifen [20]. However, some data have suggested that the positivity of ER-β is associated with low cellular differentiation, which indicates that this receptor could be related to worse overall survival [21].

Data from a number of studies comparing neoadjuvant and adjuvant endocrine treatments are now available [22]. The measurement of Ki67, a cell proliferation marker, after neoadjuvant endocrine therapy can predict the efficacy of these drugs and reflect the ability of endocrine treatment to suppress proliferation [23,24]. Indeed, Ki67 levels after 2 weeks of treatment was significantly correlated with relapse-free survival in the Arimidex, Tamoxifen, Alone or in Combination trial [23,25]. However, while the expression of ER-α has been extensively studied as a predictive marker of treatment response, the role of ER-β remains controversial and has never been examined in a neoadjuvant short-term trial.

In this context, the objective of the present study was to determine the role of ER-β and the ER-α/ER-β expression ratio in predicting the response to BC endocrine therapy with anastrozole and tamoxifen. We also focused on whether these different regimens have any effect on ER-α and ER-β expression levels. Hormone receptor proteins were detected semi-quantitatively using immunohistochemistry, and we compared the expression levels of Ki67, ER-β and ER-α before and after neoadjuvant short-term treatment in postmenopausal women with invasive carcinomas.

## Methods

### Study design, patients and treatment protocol

We designed a randomized, prospective, controlled, double-blind study that included postmenopausal women with invasive BCs.

The eligibility criteria for the study included histologically confirmed primary stage II to III invasive BC in women who were postmenopausal, which was defined as no menstruation periods over the last 12 months and/or a follicle-stimulating hormone level within the postmenopausal range. The exclusion criteria were the presence of endocrine disease, metastatic disease, inflammatory BC (T4d), history of thromboembolism and any previous treatment for BC (surgery, radio or chemotherapy). Patients who did not comply with the prescribed medication regimen or postponed surgery were also excluded from the study. Patients who had previously taken hormone replacement therapy were included if they had stopped hormonal treatment at least 6 months prior to trial randomization.

After written informed consent was obtained, 90 patients with invasive BCs were recruited into the study and enrolled at Pérola Byington Hospital and Federal University of São Paulo Hospital, Sao Paulo, Brazil, between October 2010 and May 2012. The first tumor sample was obtained from each patient at the time of diagnosis by incisional biopsy performed in an outpatient facility using local anesthesia. A second tumor specimen was obtained from each patient during definitive surgery under general anesthesia. Both tumor samples were processed using the same paraffin-embedding technique.

**Figure 1:**
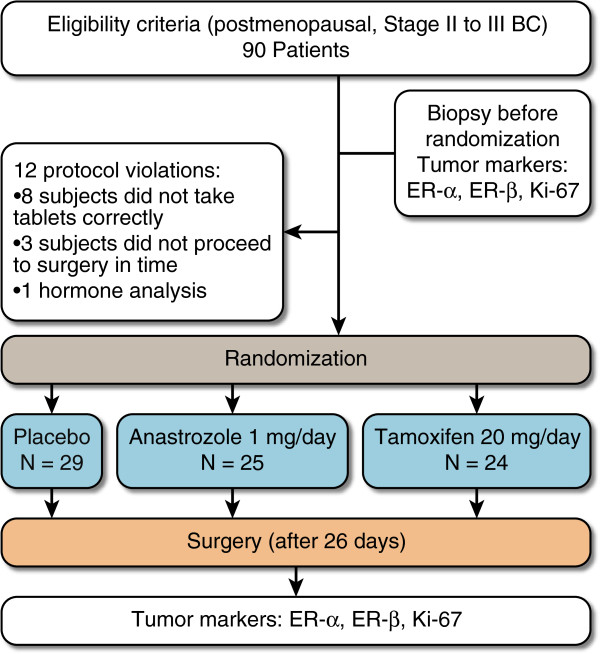
Trial schematic design.

The patients underwent definitive surgical treatment (modified radical mastectomy or conservative surgery with axillary evaluation) following a mean period of 26 days (range of 24–30 days and median of 26 days) after the incisional biopsy. There were 3 major protocol violations. These were performed by patients who did not take tablets correctly (N = 8), did not proceed to surgery in time (N = 3) or were premenopausal according to a hormone analysis (N = 1). These patients were not included in any analyses. Seventy-eight patients with operable BCs completed the study and were randomized to receive 26 days of treatment with anastrozole (N = 25) (1 mg/day), tamoxifen (N = 24) (20 mg/day) or placebo (N = 29) (Figure 1). Randomization and allocation to trial groups were carried out by a central computer system [26].

The study was approved by the Human Investigation Committees of Federal University of São Paulo and Pérola Byington Hospital under the process number CEP 0894/10, Brazil, and conducted in accordance with the Helsinki Declaration.

### Histology and tissue microarray construction

All samples were fixed in 10% neutral-buffered formalin, processed and embedded in paraffin. Respective paired tumor blocks containing samples obtained from all patients prior to any of the interventions and during definitive surgery were retrieved from the pathology files of our institution.

Specimen pairs were cut into 4-μm sections, mounted on lysine-coated slides, stained with hematoxylin and eosin and examined to confirm the diagnosis of carcinoma. The same slides were used by one pathologist investigator (AFL) to determine the area of interest (the most representative of the tumor) to be included in the tissue microarray (TMA) marked on the slide. Using a marking pen, the corresponding region was circled on the archival “donor” paraffin block. Tumor TMA blocks were obtained by punching 2-nm tissue cores from each donor paraffin block. The samples were then arrayed onto a recipient blank block using a manual tissue arrayer (Beecher Instruments, Sun Prairie, WI, USA) [27]. Control tissues were included in each of these paraffin blocks.

### Immunohistochemistry assays

After construction, 3-μm tissue sections were cut and transferred to silanized slides and then left to dry overnight at 56°C. The next day, the slides were dewaxed in xylene, rehydrated in graded alcohol solutions and washed with water. Antigen retrieval was performed using a pressure cooker (Eterna, Nigro, Sao Paulo, Brazil) and 10 mM citrate buffer, pH 6.0. The samples were quenched with 6% hydrogen peroxide and incubated overnight at 4°C with primary monoclonal antibodies for ER-α (clone SP1, Neomarkers), ER-β (clone 14C8, GeneTex) and Ki67 (clone MIB-1, DAKO). The following day, the slides were rinsed with phosphate-buffered saline (PBS) and incubated with the secondary antibody (biotinylated goat anti-mouse/rabbit immunoglobulin) diluted 1:200 for 30 min at 37°C. The slides were rinsed again with PBS and incubated with streptavidin-biotinylated-peroxidase complex (1:200, Duet mouse/rabbit horseradish peroxidase, cat No. 0492; DakoCytomation, Carpinteria CA, USA) for 30 min at 37°C. The slides were developed with 0.06% diaminobenzene as the chromogen with 0.06% hydrogen peroxide and counterstained with Harris’ hematoxylin [27]. Positive and negative control slides were included.

### Biomarker scoring

The results of immunohistochemistry were assessed by 2 investigators (AFL and MM) in a blinded fashion, independently examining the whole slide. In most cases (kappa coefficient = 0.78), the estimations of the 2 investigators were identical, and discrepancies were resolved by joint review of the slides. All slides were examined and scored semi-quantitatively according to Allred’s criteria using 2 parameters: the proportion of positive cells and the staining intensity. These parameters were independently recorded for each immunohistochemical reaction. The distribution of the proportional fraction of stained cells on each slide was scored using a scale from 0 to 5. The intensity of staining was scored from 0 to 3. The sum of these 2 partial scores resulted in a final score. Zero on this scale indicated that no cells were stained, and scores ranging from 2 to 8 indicated different levels of scoring. All cases with a final score equal to or greater than 3 were considered positive [28,29].

### Statistical analysis

The statistical analysis was conducted by an independent statistician. The hormone therapy for each patient was coded to maintain the blind assessment and avoid bias. The analytical process used the IBM SPSS Statistics 19 software (IBM, USA).

Descriptive statistics were used to summarize the sample characteristics at baseline (mean, standard deviation, minimum, median and maximum). The number of valid observations was used to summarize the numeric variables, and frequency and percentage were used to summarize the categorical variables. The groups were tested for homoscedasticity, also known as homogeneity of variance.

The changes in the ER-β scores over time among the groups (anastrozole, tamoxifen and placebo) were evaluated with an ANOVA with repeated measures using rank transformation.

The changes in the Ki67 scores over time and differences among groups (anastrozole, tamoxifen and placebo) were evaluated with an ANOVA with repeated measures for the ER-β-positive and ER-β-negative cases. To investigate whether a correlation between ER-α and ER-β existed, we calculated the Spearman’s correlation coefficient, and graphs of the expression level of each receptor were constructed.

The changes in the Ki67 scores over time and among groups (anastrozole, tamoxifen and placebo) were evaluated for different ER-α/ER-β expression ratios with an ANOVA with repeated measures using rank transformation.

The Bonferroni correction (a multiple-comparison test) was used to adjust the *p* values for multiple testing. All tests were performed with a significance level of 0.05 (p ≤ 0.05).

## Results

A total of 78 patients were included in our analyses. The statistical analysis showed that there were no significant differences in clinical characteristics between groups (age, age at first menstrual period, age at menopause, number of children, age at birth of first child or tumor size; all p > 0.10); therefore the sample was considered homogeneous. The mean age of the patients included in the study was 65.7 years, with a range of 42–89 years and median of 67 years. The mean age at menopause was 48 years, with a range of 32–60 years and median of 50 years. The average tumor size was 3.9 cm, with a range of 2.5–8.0 cm and median of 4.0 cm. The majority of patients had stage II carcinoma.

**Figure 2:**
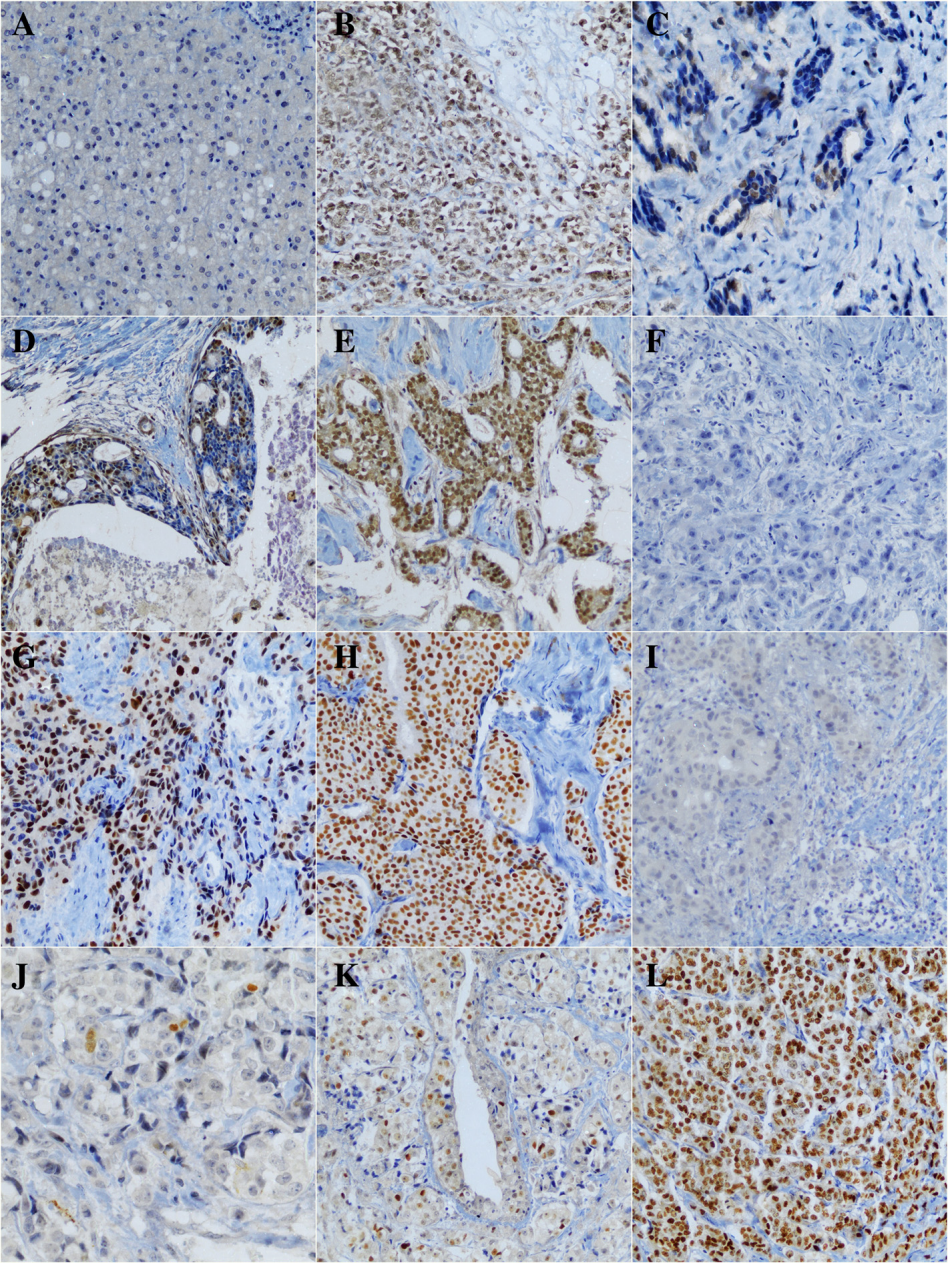
**Immunohistochemical staining pattern in BC samples for ER-β, ER-α and Ki67. A-E** examples of ER-β expression: liver as negative control **(A)**, whole section positive control slide **(B)**, Allred score 3 **(C)**, Allred score 5 **(D)** and Allred score 8 **(E)**. **F-H** examples of ER-α expression: Allred score 0 **(F)**, Allred score 6 **(G)** and Allred score 8 **(H)**. **I-L** examples of Ki67 expression; Allred score 0 **(I)**, Allred score 2 **(J)**, Allred score 5 **(K)** and Allred score 8 **(L)**.

Three tumor samples obtained at the time of diagnosis and/or during definitive surgery had insufficient invasive cancer in the biopsy when re-cut for the ER-β study, resulting in a final number of 75 patients for the receptor analysis. Examples of immunoreactivity for ER-β, ER-α and Ki67 are shown in Figure 2. The mean pre- and post-treatment Allred scores for ER-β are presented in Table 1. The frequency of ER-β expression did not change after treatment (p = 0.33).

**Table 1:** Changes of ER-β scores between treatment groups

	**A**	**P**	**T**	**Total**
Pre-treatment				
Mean ± SD	3.21 ± 2.81	3.21 ± 2.63	4.17 ± 2.57	3.51 ± 2.67
Median (minimum–maximum)	3 (0–8)	4 (0–7)	5 (0–8)	4 (0–8)
Total (n)	24	28	23	75
Post-treatment				
Mean ± SD	4 ± 2.31	2.93 ± 2.45	5 ± 2.04	3.91 ± 2.41
Median (minimum–maximum)	4 (0–7)	4 (0–7)	6 (0–8)	4 (0–8)
Total (n)	25	28	23	76

A: anastrozole group; P: placebo group; T: tamoxifen group; SD: standard deviation.

ANOVA with repeated measures using rank transformation: treatment (p = 0.3312) and group × treatment (p = 0.3052).

The distribution of patients in each study group and among randomized treatments as well as the number of ER-α-positive cases are presented in Table 2.

**Table 2:** Distribution of patients in the study groups and among randomized treatments

	**A (pre-treatment)**		**P (pre-treatment)**		**T (pre-treatment)**		**Total**	
	**n**	**ER-α-positive**	**n**	**ER-α-positive**	**n**	**ER-α-positive**	**n**	**ER-α-positive**
ER- β-negative	11	9	12	9	5	3	28	21
ER- β-positive	13	9	16	14	18	13	47	36
ER-α/ER-β ratio < 1	5	2	5	2	8	4	18	8
ER-α/ER-β ratio between 1 and 1.5	9	7	7	6	6	5	22	18
ER-α/ER-β ratio > 1.5	10	9	16	15	9	7	35	31

A: anastrozole group; P: placebo group; T: tamoxifen group.

ER-α-positive: number of patients in each group considered positive to ER-α (final Allred score equal to or greater than 3).

**Figure 3:**
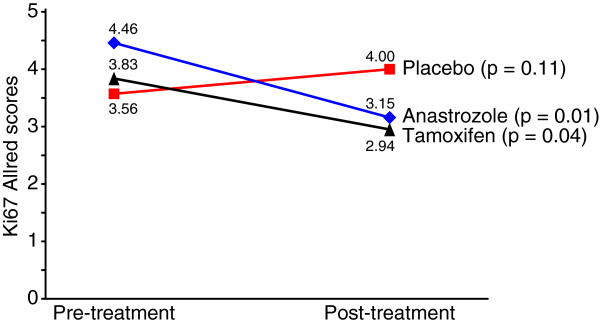
Changes in Ki67 after short-term treatment of ER-β-positive breast cancer.

There was not a significant change of Ki67 levels during neoadjuvant treatment in ER-β-negative cases (p = 0.45). In these patients, the mean pre- and post-treatment Ki67 scores were 2.3 and 2.2 in the placebo group, 4.2 and 3.5 in the anastrozole group and 4.6 and 3.4 in the tamoxifen group, respectively (Table 3). However, in the ER-β positive cases, the anastrozole group (p = 0.01) and tamoxifen group (p = 0.04) presented a significant reduction in post-treatment Ki67 Allred scores compared with baseline. In these cases, the mean pre- and post-treatment Ki67 scores were 3.6 and 4.0 in the placebo group, 4.5 and 3.2 in the anastrozole group and 3.8 and 2.9 in the tamoxifen group, respectively (Table 3 and Figure 3).

**Table 3:** Allred scores of Ki67 biomarker immunohistochemistry results in ER-β-negative and ER-β-positive cases

**Ki67**	**Anastrozole (A)**		**Placebo (P)**		**Tamoxifen (T)**	
	**Pre-treatment**	**Post-treatment**	**Pre-treatment**	**Post-treatment**	**Pre-treatment**	**Post-treatment**
ER- β-negative						
Mean ± SD	4.2 ± 1.9	3.5 ± 2.8	2.3 ± 1.4	2.2 ± 1.7	4.6 ± 1.9	3.4 ± 2.7
Median (minimum–maximum)	4 (2–8)	2 (0–8)	2 (0–4)	2 (0–5)	4 (2–7)	3 (0–7)
Total (n)	11	11	12	12	5	5
ER- β-positive						
Mean ± SD	4.5 ± 2	3.2 ± 2.1*	3.6 ± 1.8	4 ± 1.6	3.8 ± 1.8	2.9 ± 2.1**
Median (minimum–maximum)	4 (0–7)	2 (0–7)	4 (0–6)	4 (2–6)	4 (0–6)	3 (0–7)
Total (n)	13	13	16	16	18	18

SD: standard deviation.

ER-β-negative: ANOVA with repeated measures: group (p = 0.071), treatment (p = 0.084) and group × treatment (p = 0.446).

ER-β-positive: ANOVA with repeated measures: group (p = 0.726), treatment (p = 0.036) and group × treatment (p = 0.032).

Bonferroni multiple comparison test for ER-β-positive analysis: group A (pre- × post-treatment: p = 0.014*), group P (pre- × post-treatment: p = 0.113) and group T (pre- × post-treatment: p = 0.046**).

Fifty-seven of 78 cases were positive for ER-α (Table 2). The Spearman’s correlation coefficients indicated a weak but positive correlation between ER-α and ER-β (r = 0.21, p = 0.08 in pretreatment and r = 0.25, p = 0.03 in post-treatment). Eleven of 47 ER-β-positive cases were negative for ER-α. Unfortunately, the number of cases for each subdivision (groups and according to positive or negative hormone receptors ER-α and ER-β) was relatively small especially for the ER-β-negative and ER-α-negative cases, which prevented a separate statistical analysis of Ki67 variation after treatment in each group.

**Figure 4:**
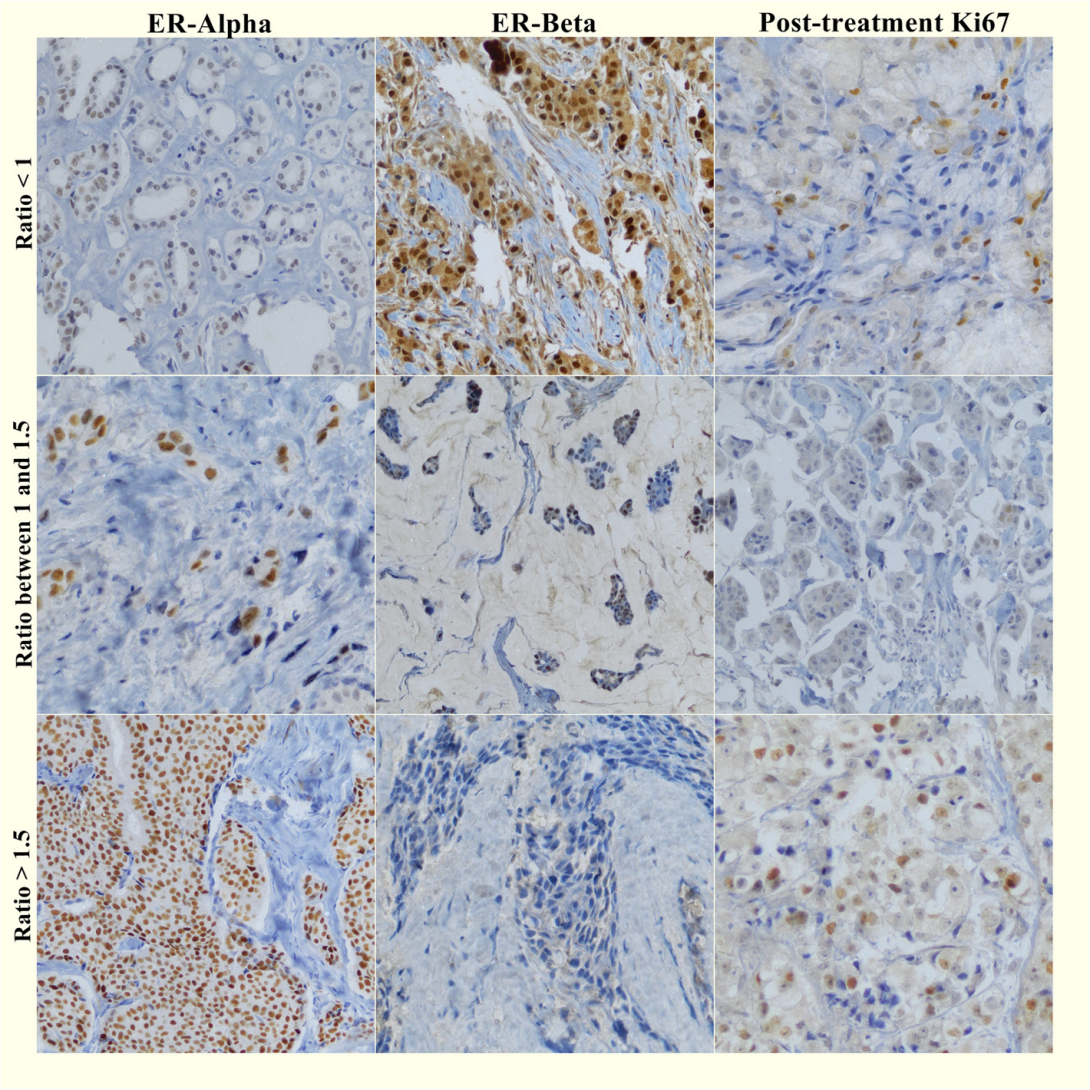
**Pretreatment Allred scores ratios of ER-α/ER-β and post-treatment Ki67 expression.** Immunohistochemical staining showing different examples of ER-α/ER-β ratios: Case 2 (ratio < 1) with a poor variation in Ki67 level; Case 37 (ratio between 1 and 1.5) with a significant difference in Ki67 level after treatment and Case 28 (ratio > 1.5) with no significant change in Ki67 expression.

We calculated the ratio of the ER-α/ER-β pre-treatment Allred scores and subdivided these patients in 3 groups: ratio < 1 (patients with a much higher ER-β than ER-α score), ratio between 1 and 1.5 (patients with similar scores between the 2 receptors and a slightly higher ER-α score) and ratio > 1.5 (patients with a much higher ER-α than ER-β score). If the denominator (ER-β score) of the fraction was zero, we considered as ratio > 1.5 (patients with a much higher ER-α than ER-β score). The exception was when the numerator (ER-α) was zero too. In this case (ER-α = zero and ER-β = zero), we considered as ratio < 1. Examples of pretreatment ER-α/ER-β ratios and post-treatment Ki67 are shown in Figure 4.

**Figure 5:**
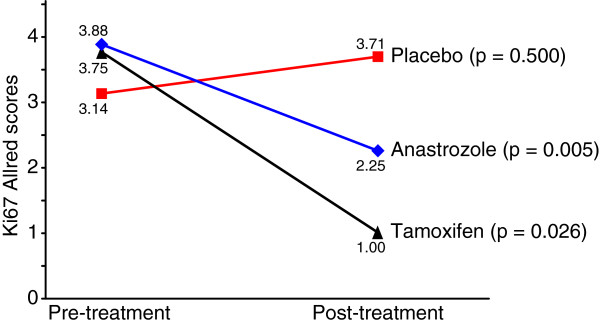
Ki67 Biomarker in patients with an ER-α/ER-β score ratio between 1 and 1.5.

After short-term treatment, there were no significant changes in Ki67 levels in the ratio < 1 (p = 0.30) and ratio > 1.5 (p = 0.41) cases. In patients with higher ER-β than ER-α scores (ratio < 1), the mean pre- and post-treatment Ki67 scores were 4.0 and 4.8 in the placebo group, 5.8 and 4.6 in the anastrozole group and 3.8 and 3.5 in the tamoxifen group, respectively. In patients with much higher ER-α than ER-β scores (ratio > 1.5), the mean pre- and post-treatment Ki67 scores were 2.7 and 2.6 in the placebo group, 4.0 and 3.5 in the anastrozole group and 4.3 and 3.4 in the tamoxifen group, respectively. However, the patients with an ER-α/ER-β score ratio between 1 and 1.5 demonstrated significant differences in Ki67 levels after treatment. For the anastrozole (p = 0.005) and tamoxifen (p = 0.026) groups, the Ki67 score was significantly lower after treatment compared with the first biopsy Ki67 score (Figure 5).

## Discussion

The development of new treatments and the assessment of biomarkers to improve BC patient outcomes require very large randomized adjuvant clinical trials that may extend over several years before the first results are available. Neoadjuvant studies provide an opportunity to integrate the molecular determinants of response and resistance with the clinical response of primary BC to medical therapy [23,30,31].

The optimum time to evaluate biomarkers for tumor response (apoptosis and proliferation) is not defined. Although cellular changes have been described *in vitro* after 24 hours of drug exposure, Dowsett *et al*[23] reported that after two weeks of neoadjuvant treatment of primary breast cancer with anastrozole and tamoxifen, cellular changes are similar to those observed after 12 weeks of treatment. As other similar studies [23,25,28,30], the classical dose of tamoxifen (20 mg/day) is enough to reach steady state after 14 days of short-term treatment. The period of 26 days was chosen because this is the average time needed to complete routine preoperative testing in our institutions, justifying the inclusion of ER-α-negative patients and the use of placebo without ill consequences to the ER-α-positive patients.

Although there is no consensus, the clinicopathologic importance of ER-β expression in BC is emerging, including its connection with factors usually associated with a better clinical outcome [11,17,32,33]. Until now, data about these favorable prognoses were based on protein studies in BC tissues and cellular experiments [34] or retrospective studies that have assessed ER-β expression in relation with the clinical outcome associated with endocrine therapy in BC [35]. In the present study, ER-β expression did not change with exposure to any of the tested drugs, but ER-β-positive postmenopausal patients treated with anastrozole and tamoxifen presented a significant reduction of Ki67 expression after neoadjuvant short-term treatment.

Post-treatment ER-β expression did not vary significantly between the 3 groups. This is similar to ER-α expression, which did not vary significantly, as reported in the Immediate Preoperative Anastrozole, Tamoxifen, or Combined with Tamoxifen trial [25] and in more recent studies [28]. Thus, post-treatment ER-β expression alone does not appear to be an early predictor of response to short-term anastrozole and tamoxifen therapies. In a randomized trial of vorozole versus tamoxifen [36], there was a decrease in ER-α expression with both drugs, and this has also been found in a study comparing letrozole and tamoxifen [24]. However, in stimulation assays, Smollich *et al*[37] indicated that tamoxifen and fulvestrant increased ER-α expression and left ER-β expression unchanged, while AI up-regulated ER-β (anastrozole, p = 0.029; letrozole, p = 0.048). These data indicate that SERMs/antiestrogens and AI can exhibit opposing effects on the ER expression of BC cells, which may contribute to the therapeutic superiority of AI over antiestrogens. Interestingly, it has been found that ER-β is significantly up-regulated, whereas ER-α is down-regulated in tumors after treatment of premenopausal women with BCs with adjuvant letrozole in combination with gonadotropin-releasing hormone (GnRH) analogues [38]. In addition, patients treated with anastrozole but not with tamoxifen have a significant reduction in PgR expression [25,28,29]. It is likely that the production of estrogen is consistently blocked and that the expression PgR is significantly reduced by the action of AI.

Short-term changes in Ki67 are not intended to be used for treatment decisions in individual patients. However, they do support the use of this clinical model for the evaluation of new agents before the initiation of large-scale adjuvant trials. Independently of ER-α status, the results from our prospective study demonstrate that ER-β- positive BC treated with anastrozole and tamoxifen presents a significant reduction in Ki67 expression after neoadjuvant short-term treatment compared with placebo and ER-β-negative cases. In a 58 ER-α-positive BC patient study, Mattar *et al*[28] demonstrated that short-term tamoxifen therapy was not associated with a significant reduction in Ki67 expression. However, some important studies have demonstrated paradoxical Ki67 increases after neoadjuvant endocrine therapy [23,24,39]. Ellis *et al*[24] observed an increase in Ki67 with treatment in HER1/2-negative cases. The molecular basis for this advantage appears complex but includes a possible tamoxifen agonist effect in ER-α-positive BC. In addition, the degree of Ki67 suppression varies markedly between tumors in some trials [25], and this indicates that the degree of estrogenic dependence is highly variable between tumors.

Our data indicate that ER-β positivity could predict the tamoxifen effect in BC treatment with no initial increase of Ki67 (the tamoxifen flare phenomenon). In fact, there is substantial evidence for ER-β as a predictor of the tamoxifen endocrine response [17,40,41]. Recently, Yan *et al*[42] analyzed ER-β and its co-regulator Steroid Receptor RNA Activator Protein (SRAP) expression in tissue microarrays from a randomized, placebo-controlled trial and found that the benefit was only in the tamoxifen-treated but not in the placebo arm; therefore providing evidence that ER-β expression was predictive for response to tamoxifen inhibition of tumor growth and survival particularly in ER-α-negative premenopausal early BC. Another study indicated that ER-β enhances the antiestrogenic actions of endoxifen in BC cells [43]. Thus, the potential benefit from tamoxifen therapy observed in our clinical study with patients whose tumors are ER-β-positive may be mediated through the actions of endoxifen. In addition, the ability of low endoxifen concentrations to significantly inhibit estrogen-induced gene expression and proliferation in ER-β-expressing BC cells suggests that the benefits from tamoxifen therapy may still be observed in patients characterized as poor metabolizers based on their Cytochrome P450 2D6 genotype if their tumors are ER-β-positive [43]. Tamoxifen is an effective drug, but 2 drawbacks are associated with its clinical use: not all ER-α-positive BCs respond to tamoxifen, and most patients develop resistance to tamoxifen with prolonged use. Given recent insights into the understanding of estrogen signaling and how ER-β is involved, these negative aspects of tamoxifen can be understood, and better methods for testing cancers for sensitivity to tamoxifen and for the development of tamoxifen resistance are available [44]. The assessment of pretreatment ER-β phenotype and changes in that phenotype with therapy alongside the changes in Ki67, as observed in our data, may help establish the mechanisms that contribute to the variable response observed and lead to strategies that may overcome tamoxifen resistance.

The most important question for clinicians is whether the ER-β status provides clinically useful information over what is already provided by the traditional ER-α/PgR assay. On this matter, there are 2 groups of BCs, one in which ER-β is coexpressed with ER-α and the other in which ER-β is expressed alone. The first group comprises approximately 59% of all primary BCs, while the ER-β alone-expressing group comprises approximately 17% [34]. Promising findings in ER-β-positive/ER-α-negative BC cases have demonstrated that ER-β status is a significant prognostic factor in univariate and multivariate analysis [17]. In this study based on the archival material of 442 BCs from women treated with adjuvant tamoxifen, ER-β positivity in ER-α/PgR-negatives cases was associated with significantly better survival compared with ER-β negative BC. In ER-α-negative tumors, ER-β expression appears to be associated with longer disease-free survival upon endocrine treatment [41]. Some findings also indicate the possibility that ER-β expression levels might be especially relevant for prognostic stratification of the ER-α-positive/PgR-positive tumors, which have a more favorable natural history [45].

Shortly after the discovery of ER-β, it was shown that ER-β mediates other and opposite effects to those of ER-α [46]. Upon ligand activation, the receptors dissociate, change conformation and form functional dimers at specific DNA elements. Depending on the presence of ER-α and ER-β in a certain cell, the receptors form functional homo- or heterodimers on promoter elements [40]. ER-β appears to reduce the cell proliferation induced by ER-α activation. Since the first evidence that ER-β is an important modulator of proliferation and invasion of BC cells, it has been shown that the ratio of ER-α/ER-β expression is higher in BC than normal tissues due to the lower expression of ER-β, supporting the hypothesis first shown by League *et al*[47] that the loss of ER-β expression could be one of the events leading to the development of BC tumorigenesis. The reason for this loss of ER-β in cancer appears to be the silencing of ER-β via promoter methylation [44,48].

The identification of five major variants of ER-β (β1, β2/cx, β3, β4 and β5), mainly generated through alternative splicing events, increases the complexity of interpreting the literature data accumulated using only one antibody for immunodetection of ER-β expression [49,50]. There is no consensus regarding the function of each variant and contradictory results concerning potential function have been published [51]. It seems that the variant ER-β isoforms can modify both ER-α and ER-β1 activity when co-expressed. Therefore, differential expression of the ER-β variants may play a role in the so-called bi-faceted ER-β action and sensitivity to antiestrogens during breast tumorigenesis and breast cancer progression [49]. Our immunostainings were carried out using a monoclonal anti-ER-β antibody (clone 14C8 from GeneTex), which is pan-specific for ER-β isoforms. Therefore, we evaluated total ER-β protein levels by performing immunohistochemistry using this well-characterized antibody, previously shown to be one of the best-performing antibodies for this application [52].

Our data also indicate a weak but positive correlation between ER-α and ER-β and demonstrate significant decreases in Ki67 levels after treatment with both anastrozole and tamoxifen only in patients with a ratio of ER-α/ER-β Allred scores between 1 and 1.5. No changes in Ki67 levels were observed in patients with higher ER-β than ER-α scores (ratio < 1) or with much higher ER-α scores than ER-β (ratio > 1.5). The effects of hormonal treatment on cell proliferation are apparently dependent on the actual ratio of ER-α/ER-β expression levels in these tumors and not only the receptor positivity. Sotoca *et al*[53] investigated how variable cellular expression ratios of ER-α and ER-β modulate the effects on cell proliferation induced by ER-α or ER-β agonists, respectively. Consistent with our results, they found the use of ER-β protein expression levels as a biomarker in tumor screening, in addition to protein expression levels of ER-α, to be a more successful indication of therapeutic responses and course/outcome of the disease in ER-positive tumors [53]. In fact, *in vitro* studies have indicated that a tamoxifen treatment of ER-α BC cells has an even stronger effect in the presence of ER-β [12]. Because ER-α and ER-β differ in affinity for promoter elements, this might explain their difference in tamoxifen responses. Estrogen response element activity is repressed by both ER-α and ER-β in the presence of tamoxifen, while activator protein 1 (AP-1) responsive elements are activated by both receptors in the presence of tamoxifen. When ER-β is expressed in parallel with ER-α, which is the case of our patients with a ratio of ER-α/ER-β Allred scores between 1 and 1.5, the activation of AP-1 elements is inhibited by ER-β [40,54], and this could play an important role in the behavior of BC cells in response to tamoxifen. The role of ER-β in response to AI therapy is unclear. In a study by Torrisi *et al*[38], it was found that ER-β is significantly up-regulated, whereas ER-α is down-regulated after treatment of 32 premenopausal women with BCs with adjuvant letrozole in combination with a GnRH analogue. Our study with postmenopausal women treated with anastrozole also demonstrated a decrease in Ki67 levels after treatment with anastrozole only in patients with a ratio of ER-α/ER-β Allred scores between 1 and 1.5. It is possible that ER-β or its relationship with ER-α is important in the therapeutic response to AI.

These results support the hypothesis of other authors [35] who have suggested that the assessment of ER-β together with ER-α is a better predictor of endocrine responsiveness than ER-α alone. In addition, as some studies have suggested that ER-β correlates with and regulates PgR expression together with ER-α [55-57], it is possible that ER-β and ER-α could be better biomarkers than ER-α and PgR. It is also possible that the 3 receptors in combination may provide the most precise prediction of endocrine responsiveness.

Our study was hampered by relatively small sample size. The number of cases according to positive or negative hormone receptors (especially for the ER-β-negative and ER-α-negative cases) prevented a separate statistical analysis of Ki67 changes after treatment in each group. A systematic and larger study, taking ER-β status into consideration, for patients with different positivity for receptors (ER-β, ER-α and PgR) could better characterize each cancer and help to optimize adjuvant treatment for BC patients. Some differences of our conclusions compared with other studies should be drawn keeping in mind the large amount of ER-β antibodies used in the literature and the various cut points for determining the positivity of ER-α and ER-β. Some published data on the usefulness of several ER-β antibodies for a number of analyses including immunohistochemistry have underscored the marked variations in specificity and likely sensitivity that exist for the different antibodies currently available [58]. In addition, our Brazilian population is one of the most heterogeneous in the world, formed mainly by the admixture between European, African and Native American populations and, more recently, individuals of Asian origins. These race-specific factors may also influence our findings compared with the white population of others studies. Although no studies have examined specifically differences in ER-β protein expression with regards to ethnicity, two studies showed that ER-β mRNA levels are significantly decreased in ER-α-positive BC from African American women [59] and from East Asian women [60]. It should also be noted that the patients enrolled onto this trial represent only a small percentage of our whole postmenopausal BC population treated in our institutions during the entry period. Several studies failed to find significant correlations between ER-β expression and patient age [11], however, it may be considered another limitation of our study.

## Conclusions

Our results demonstrated for the first time for neoadjuvant short-term treatment that ER-β expression did not change during endocrine treatment and may predict the effects of anastrozole and tamoxifen in postmenopausal BC patients. These effects of hormonal treatment on cell proliferation appear to be dependent on the ratio of ER-α/ER-β expression. This study supports further investigation into whether ER-β could be a predictor of endocrine responsiveness or whether the receptor could be used as a target in selected groups of BC.

## Abbreviations

BC: Breast cancer; ER: Estrogen receptor; ER-α: Alpha estrogen receptor; PgR: Progesterone receptor; SERM: Selective estrogen-receptor modulator; AI: Aromatase inhibitor; ER-β: Beta estrogen receptor; TMA: Tissue microarray; PBS: Phosphate-buffered saline; GnRH: Gonadotropin-releasing hormone; SRAP: Steroid Receptor RNA Activator Protein; AP-1: Activator protein 1.

## Competing interests

The authors declare no competing interests.

## Authors’ contributions

MM contributed to the acquisition of the data, biomarker scoring, analysis/interpretation of the data, and discussions of the content of the manuscript, composition of the manuscript, and revisions to the manuscript before submission. AM participated in the conception, design, acquisition and analysis/interpretation of data. AFL performed the histology and tissue microarray construction and contributed to biomarker scoring and data interpretation. FAS performed histology and Immunohistochemistry assays. LHG conceived the study and contributed to the study design, study coordination, data analysis/interpretation and manuscript composition. All authors read and approved the final manuscript.

## Pre-publication history

The pre-publication history for this paper can be accessed here:

http://www.biomedcentral.com/1471-2407/13/425/prepub
